# Integrating Phylogenetic and Morphological Data Reveals New Taxa of *Hydnum* (*Hydnaceae*, *Cantharellales*) from China

**DOI:** 10.3390/jof12090674

**Published:** 2026-09-09

**Authors:** Ting Cao, Dong-Xu Ji, Hai-Sheng Yuan, Tie-Zhi Liu

**Affiliations:** 1 Key Laboratory of Environmental Pollution and Microecology of Liaoning Province, Shenyang Medical College, Shenyang 110034, China; jidongxu@symc.edu.cn; 2 CAS Key Laboratory of Forest Ecology and Silviculture, Institute of Applied Ecology, Chinese Academy of Sciences, Shenyang 110164, China; hsyuan@iae.ac.cn; 3 College of Chemistry and Life Sciences, Chifeng University, Chifeng 024000, China; tiezhiliu@aliyun.com

**Keywords:** *Hydnaceae*, *Hydnum*, multiple-marker phylogeny, new taxon, taxonomy

## Abstract

*Hydnum* is a genus of ectomycorrhizal fungi characterized by umbrella-shaped basidiocarps and hydnoid hymenium in the family *Hydnaceae* of *Cantharellales*. New species of this genus have been increasingly discovered worldwide, particularly in Asia in recent years. But compared with other fungal groups, the species richness of *Hydnum* still warrants sustained attention. In this study, phylogenetic analyses of *Hydnum* in China were conducted based on the dataset of the large subunit of the nuclear ribosomal RNA gene (nLSU), internal transcribed spacer regions (ITS) and translation elongation factor 1-alpha gene (*tef*-*1α*). Maximum Likelihood and Bayesian analyses were used to reconstruct the phylogenetic tree. Five phylogenetic taxa have been recognized and described or redescribed employing phylogenetic and morphological analyses: one new subsection, namely the *Hydnum* subsect. *Tangerinum*, two new species namely *H. cavum* and *H. dazhouense*, as well as two newly recorded species of China: *H. albopallidum* and *H. repando-orientale*. A key to the species of *Hydnum* in China was provided.

## 1. Introduction

*Hydnum* L. is typified by *H. repandum* L. [1] and was established by Linnaeus in 1753 [2]. Morphologically, the species in *Hydnum* were traditionally characterized by pileate basidiocarps, aculeate hymenophore, stichic basidia, azoned monomitic context, smooth and subglobose to obovoid-elliptic basidiospores and usually the absence of cystidia [1,3,4]. Kreisel [5] placed the genus in *Cantharellales* Gäum based on its stichic basidia and this was confirmed by several subsequent molecular analyses [2,6,7,8]. Cao et al. [9] conducted a comprehensive multi-marker phylogenetic analysis which confirmed 17 genera within *Hydnaceae*. As the type genus of *Hydnaceae*, *Hydnum,* is the sister clade of *Sistotrema confluens-subconfluens* lineage and closely related to *Cantharellus* Lam. as well as *Craterellus* Pers. [2,9].

For the genus *Hydnum*, Grebenc et al. [10] conducted a comprehensive molecular phylogenetic study of European *Hydnum* and identified ten distinct lineages. Olariaga et al. [11] expanded upon the work of Grebenc et al. by describing four new species from North America and Europe, and formally described one lineage RE2 as *H. vesterholtii* Olariaga, Grebenc, Salcedo & M.P. Martín. Vizzini et al. [3] described RU3 as *H. magnorufescens* Vizzini, Picillo & Contu, while Yanaga et al. [12] reported three new *Hydnum* species from Japan. In 2016, Feng et al. classified at least 31 species of *Hydnum* worldwide into four major clades: the *Albomagnum*, *Vesterholtii*, *Rufescens*, and *Repandum* clades [13]. Then Niskanen et al. [1] recognized four subgenera, four sections and five subsections within *Hydnum*. Subsequently, as the new species are continuously discovered and the framework research progresses further, there are six subgenera, four sections, four subsections and a described clade *Insulana* within *Hydnum* [9,14,15,16,17].

The habitats of species in *Hydnum* are often closely related to a broad range of plant families, including the *Betulaceae* Gray, *Dipterocarpaceae* Blume, *Juglandaceae* DC.ex Perleb, *Fagaceae* Dumort., *Magnoliaceae* Juss., *Malvaceae* Juss., *Myrtaceae* Juss., *Pinaceae* Lindley, *Salicaceae* Mirb. and *Ulmaceae* Mirb. [1,13,18,19,20]. As typical ectomycorrhizal fungi, the species of *Hydnum* exhibits a worldwide distribution. Although the Index Fungorum database lists 982 species names for the genus, only approximately 70 species have been validly described (http://www.indexfungorum.org, accessed on 3 August 2026). Documented occurrences are primarily from Asia, Europe, and North America [11,12,14,21,22,23,24], which suggests that numerous *Hydnum* species worldwide remain poorly understood and require further exploration. To date, research on *Hydnum* has intensified in China and over 40 species have been recognized based on molecular and morphological evidence [9,13,15,16,17,25,26,27,28].

In recent years, we collected several special specimens of *Hydnum* from subtropical, temperate and cold temperate regions of China. One new subsection, two new species and two newly recorded species were described using morphological and phylogenetic analyses based on the ITS-nrLSU-*tef-1α* database in this study. The discovery of several new and newly recorded species from China has enriched the diversity of the genus *Hydnum* in forests across different climate zones in China. Additionally, we have reorganized the phylogenetic framework within the genus and updated the key for the *Hydnum* species in China.

## 2. Materials and Methods

### 2.1. Morphological Studies

The studied specimens were deposited at the herbarium of the Institute of Applied Ecology, Chinese Academy of Sciences (IFP). Nomenclature and taxonomic placement followed Index Fungorum (http://www.indexfungorum.org) and the mycological classification of Wijayawardene et al. [28]. The macroscopic descriptions were based on fresh basidiocarps in field or basidiomata photographs. Special colour terms are from Kornerup and Wanscher [29]. Microscopic procedures followed Cao et al. and Liu et al. [9,30] and were based on dried material. Tissue to be observed were stained with 1% aqueous solution of Congo Red and mounted in 3% aqueous KOH. Melzer’s reagent to test for any Amyloid and/or dextrinoid reactions (Melzer’s reagent: 1.5 g KI (potassium iodide), 0.5 g I (crystalline iodine), 22 g chloral hydrate, distilled water 20 mL). Sections were studied using a Nikon Eclipse E600 microscope (Nikon Corporation, Tokyo, Japan) up to 1000× with phase-contrast illumination and dimensions were estimated subjectively with an accuracy of 0.1 μm. The following abbreviations are used in the text: KOH = 3% potassium hydroxide; Lm = mean spore length (arithmetic average of all spores); Wm = mean spore width (arithmetic average of all spores); and Q referred to the length/width ratio of an individual basidiospore and avg. Q = Lm/Wm between specimens studied, and *n* = total number of spores measured from a given number of specimens. Microscopic drawings were made with the aid of a drawing tube. The spores’ measurements excluded the apiculus, and 5% of the measurements at each end of the range are given in parentheses.

### 2.2. DNA Extraction, PCR Amplification and Sequencing

Genomic DNA was extracted from dried herbarium specimens with a Thermo Scientific Phire Plant Direct PCR kit (Thermo Fisher Scientific, Waltham, MA, USA). PCR reactions were performed in 30 μL reaction mixtures containing 15 μL of 2 × Phire^®^ Plant PCR buffer, 0.6 μL Phire^®^ Hot Start II DNA Polymerase, 1.5 μL of each PCR primer (10 μM), 10.5 μL double deionized H_2_O (ddH_2_O), and 0.9 μL template DNA. ITS was amplified with the primers ITS1F/ITS4 [31]; LROR/LR5 [32] for partial nrLSU; and Tef1F/Tef1R [33] for *tef*-*1α* of *Hydnum*. The PCR thermal cycling program is as follows: denaturation at 95 °C for 3 min; followed by 34 cycles including denaturation at 95 °C for 40 s, annealing (at 58 °C for ITS, 53 °C for nrLSU, 50 °C for *tef*-*1α*, respectively) for 30 s, extension at 72 °C for 60 s, and a final extension at 72 °C for 8 min. PCR amplification was sequenced at the Beijing Genomics Institute (BGI). The sequences’ quality control followed the guidelines by Nilsson et al. (2012) [34]. All newly obtained sequences were compared with the BLAST tool (https://blast.ncbi.nlm.nih.gov/Blast.cgi, accessed on 3 August 2026) and submitted to GenBank [35]. Sequences for phylogeny analysis were found in GenBank (http://www.ncbi.nlm.gov) using the BLAST option or following the previous studies (Table 1). DNA alignments were performed using the MAFFT v.7.471 online service (https://mafft.cbrc.jp/alignment/server/index.html, accessed on 30 April 2026) and manually corrected using MEGA v. 7.0 [36,37].

### 2.3. Phylogenetic Analyses

We assembled a three-locus (ITS, nrLSU and *tef*-*1α*) dataset of *Hydnum* for phylogenetic analyses. The dataset was partitioned by gene and the best-fit models were determined by jModelTest 2.1.10 [38]. Phylogenetic analyses were conducted applying Maximum Likelihood methods and Bayesian analysis. All characters were weighted, and gaps were treated as missing data. Maximum Likelihood (ML) analysis was performed in RAxML v8.2.4 [39]. Bayesian analysis was with MrBayes 3.2.7 [40]. Four simultaneous Markov chains were run with 1 million generations and starting from random trees and keeping one tree for every 100th generation until the average standard deviation of split frequencies was below 0.01. The value of burn-in was set to discard 25% of trees when calculating the posterior probabilities. Bayesian posterior probabilities (BPP) were inferred from the 50% majority-rule consensus of trees sampled after burn-in. Branches with BPP > 0.90 were considered strongly supported. Phylogenetic trees were checked and modified in FigTree 1.4 [41].

## 3. Results

### 3.1. Phylogenetic Analyses

**Figure 1:**
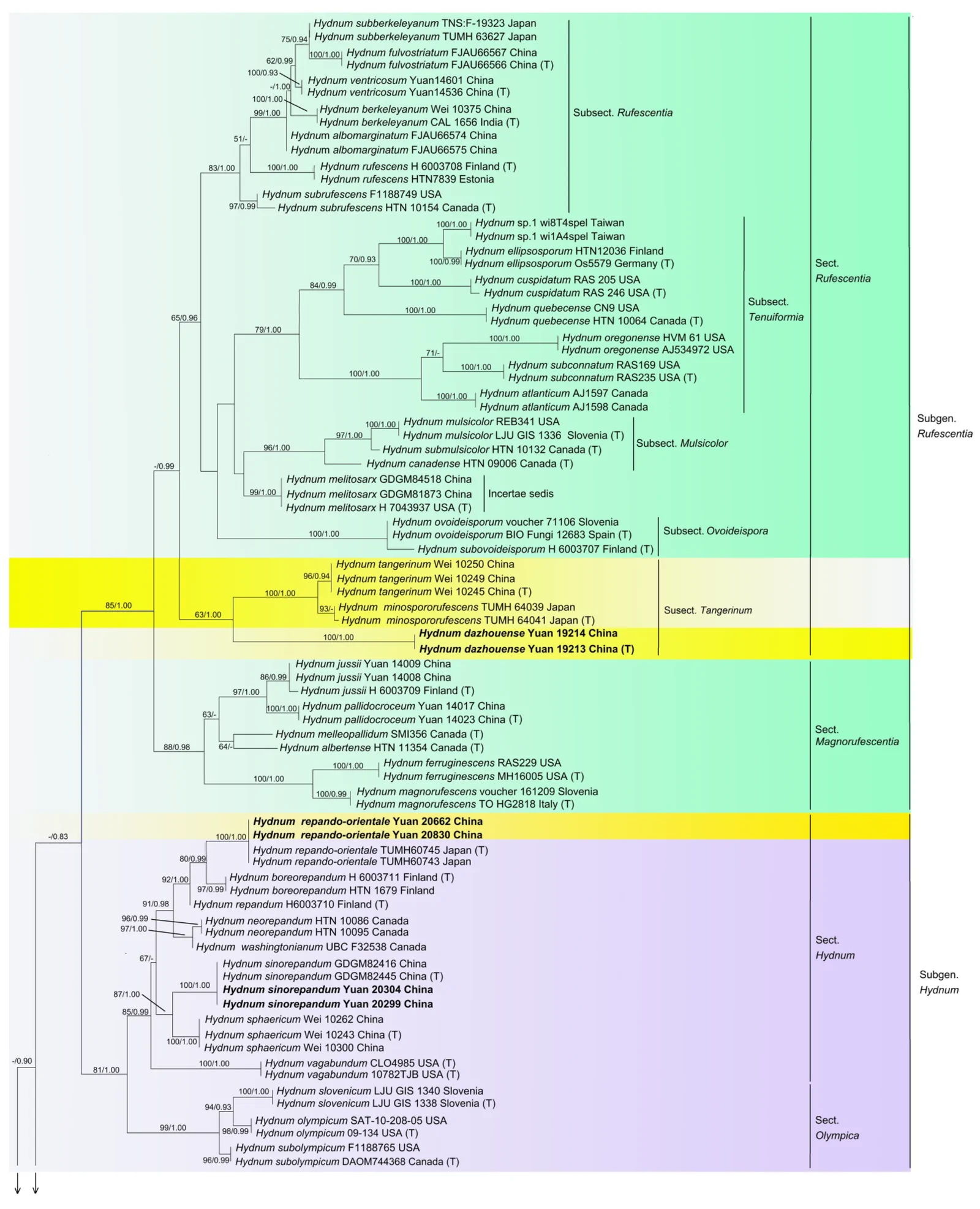

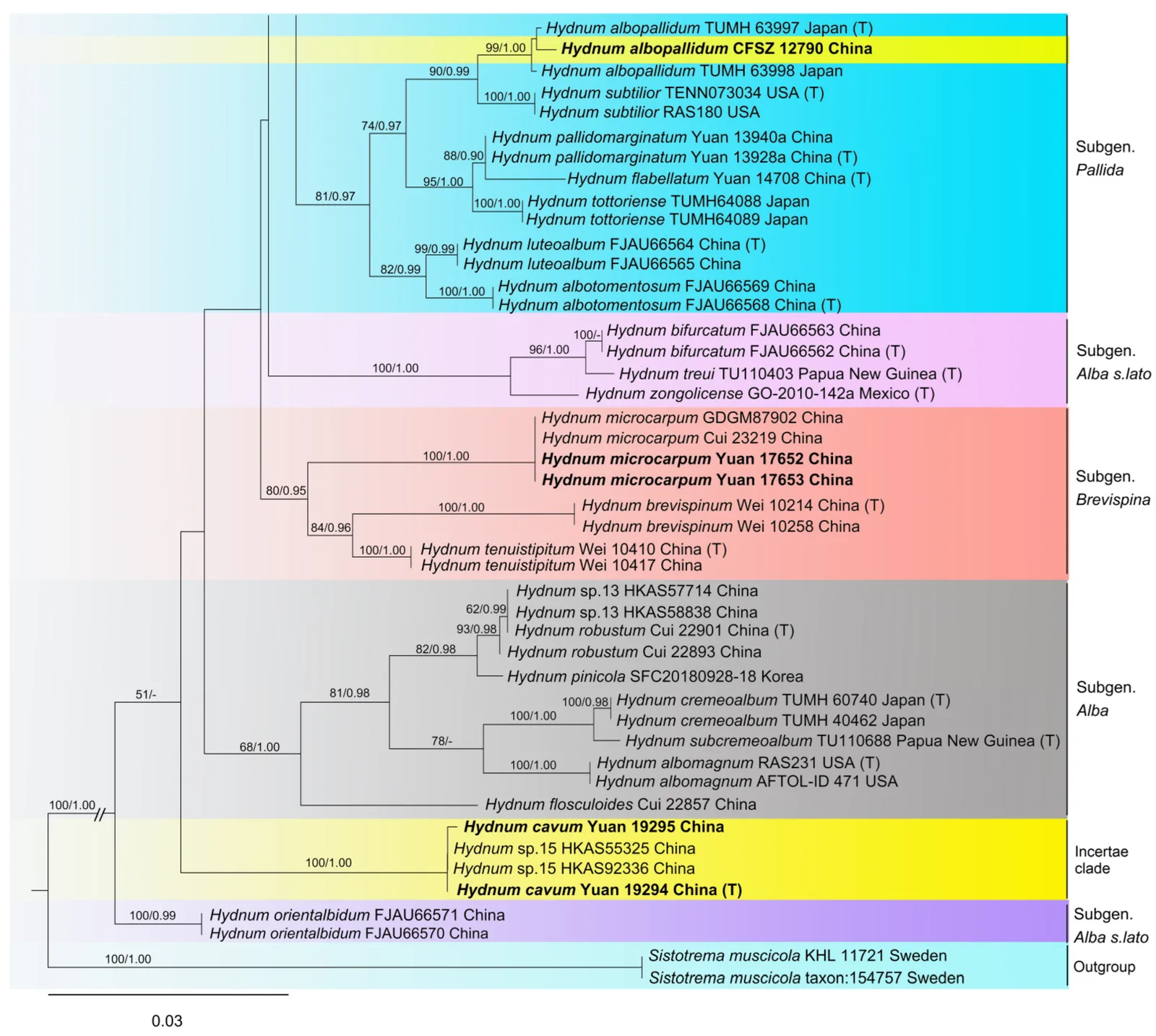
Maximum Likelihood tree based on the combined nLSU + ITS + *tef*-*1α* sequence dataset illustrating the phylogeny of the genus *Hydnum*. The new taxon has a yellow background; newly acquired samples in this study are in bold. Branches are labelled with Maximum Likelihood bootstrap higher than 70% and Bayesian Posterior Probabilities > 0.90.

26 sequences for 11 samples of *Hydnum* (2 new species in this study and 4 known species) have been newly obtained in this study. We generated a total of 126 samples which including 124 of *Hydnum* while 2 as outgroup (*Sistotrema muscicola* (Pers.) S. Lundell). The best-fit models are TIM + G for ITS, TPM1uf + I+G for nrLSU and TrNef + G for *tef*-*1α*. The final alignment comprised 1834 characters (583 for ITS, 701 for LSU and 550 for *tef*-*1α*), with 45.86% gaps and undetermined characters. The Maximum Likelihood optimization yielded a log-likelihood value of −8690.119026 (lnL). The estimated base frequencies were pi(A) = 0.2566, pi(C) = 0.1952, pi(G) = 0.2421, and pi(T) = 0.3061. ML analyses employing a GTRGAMMA substitution model and the best tree was obtained by performing 1000 rapid bootstrap inferences followed by a thorough search for the most likely tree. Bayesian analyses using a Markov Chain Monte Carlo (MCMC) algorithm and two Markov chains were run from a random starting tree and the average standard deviation of split frequencies was 0.009789. ML and Bayesian analyses have obtained similar topology and only the ML tree is shown in Figure 1 with the ML bootstrap values and Bayesian posterior probabilities.

In the phylogenetic tree, six subgenera, *Alba*, *Alba s.l.*, *Brevispina*, *Hydnum*, *Pallida* and *Rufescentia*, and two sections *Hydnum* and *Olympica,* have been confirmed with moderate to strong support. *Hydnum* subsect. *Tangerinum* T. Cao & H.S. Yuan, two new species *H. cavum* T. Cao & H.S. Yuan and *H. dazhouense* T. Cao & H.S. Yuan, two new recorded species of China *H. albopallidum* R. Sugaw. & N. Endo and *H. repando-orientale* Liimat. & Niskanen are revealed according to the analysis. The clades of two new species, *H. cavum* and *H. dazhouense,* both received full support (100% ML/1.00 BPP). *H. cavum* forms an independent branch which is distinct from any subgenus in *Hydnum*. *H. dazhouense* fell in the Subg. *Rufescentia* and grouped with *H. tangerinum* and *H. minospororufescens* R. Sugaw. & N. Endo with moderate support (63% ML/1.00 BPP).

### 3.2. Taxonomy

***Hydnum*** **subsect.** ***Tangerinum*** T. Cao & H. S.Yuan, subsect. nov., Figure 1.

MycoBank: MB 864734

Etymology: *Tangerinum* (Lat.), following the name of the type species.

Type species: *Hydnum tangerinum* T. Cao & H. S. Yuan. MycoBank MB 839421.

Note: Three species, *H. dazhouense*, *H. tangerinum* T. Cao & H.S. Yuan and *H. minospororufescens* R. Sugaw. & N. Endo comprise the new subsection and they share the following features: basidiocarps small to medium, solitary to concrescent, sometimes multipileate; pilus pale yellow, pale orange, orange to orange-brown; spines non-decurrent; basidiospores globose, subglobose to broadly ellipsoid (Q av. = 1.08–1.31). It matches the description of the descriptions of sect. Rufescentia Niskanen & Liimat by Niskanen et al. in 2018 [1,9,14]. Notably, the three species of this subsect occur in broad-leaved or mixed forests (*H. tangerinum* and *H. minospororufescens* in broad-leaved, *H. dazhouense* in mixed forest) in the subtropical zones of Asia. Phylogenetically, the new species *H. dazhouense* gathered together with *H. tangerinum* and *H. minospororufescens* formed the subsect. *tangerinum* (63% ML/1.00 BPP). The new subsect nested in sect. *Rufescentes* and grouped with subsect. *Mulsicolores*, *Ovoideispori*, *Rufescentes* and *Tenuiformia* (Figure 1). Morphologically, subsect. *Mulsicolores* can be differentiated from subsect. *Tangerinum* by having broader basidiospores (Q av. < 1.15), subsect. *Ovoideispori* and *Rufescentia* both differ by having slender basidiospores (Q av. value usually > 1.15 and >1.19, respectively) and subsect. *Tenuiformia* can also be clearly distinguished by its large and ellipsoid basidiospores (av. 10.0 × 6.6 µm). Furthermore, subsect. *Tangerinum* is closely related to sect. *Magnorufescentes*. Morphologically, three species in the new subsect. differ from species of sect. *Magnorufescentes* in having small to medium basidiomata, non-decurrent spines and globose, subglobose to broadly ellipsoid basidiospores (Q av = 1.08–1.31 vs. Q 1.06–1.13) [1].

**Figure 2:**
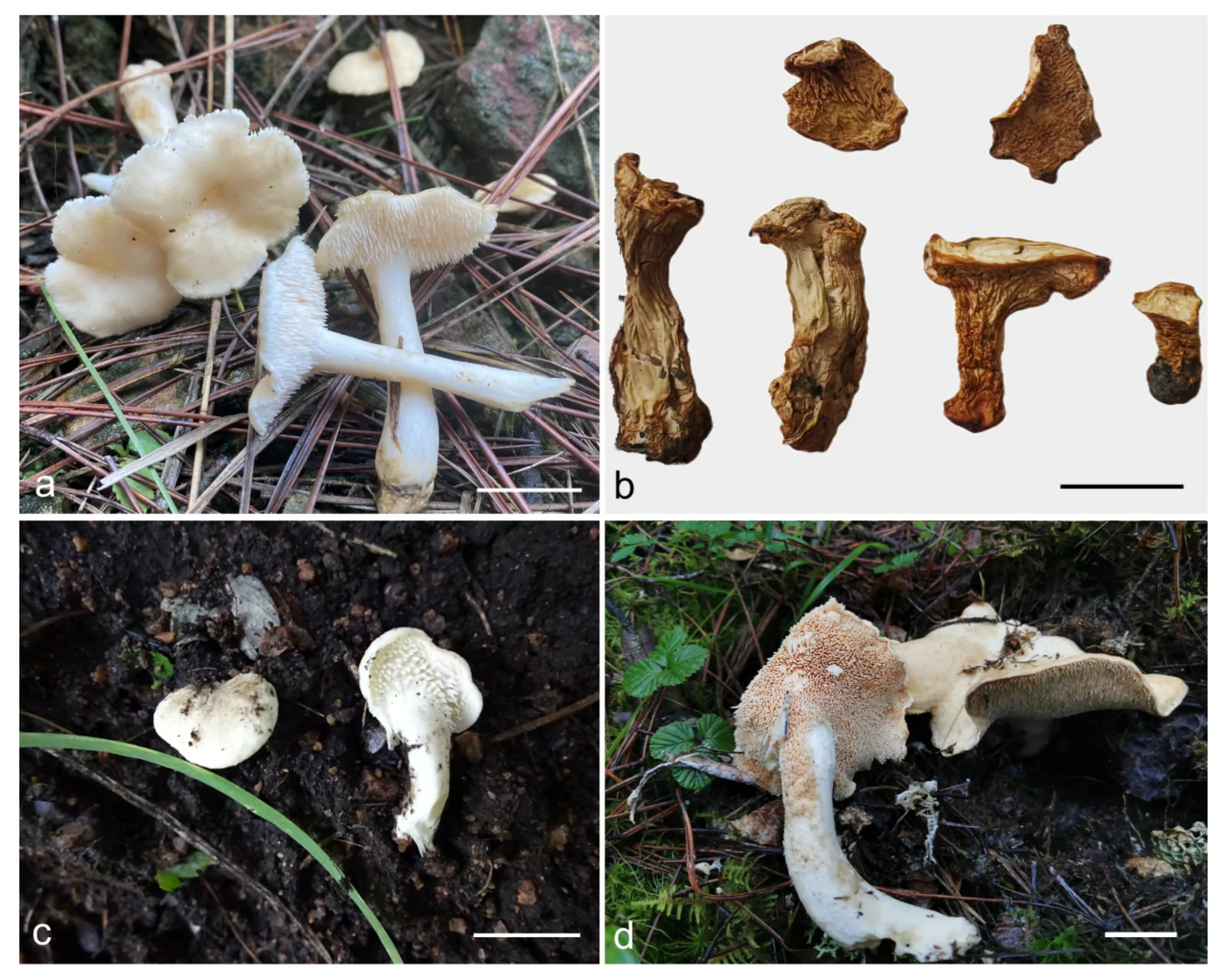
Basidiocarps of new taxa in Hydnum. (**a**) *Hydnum cavum* (holotype Yuan 19294); (**b**) *Hydnum dazhouense* (holotype Yuan 19213); (**c**) *Hydnum albopallidum* (CFSZ 12790); (**d**) *Hydnum repando-orientale* (Yuan 20830). Scale bars: (**a**–**d**) = 1 cm.

**Figure 3:**
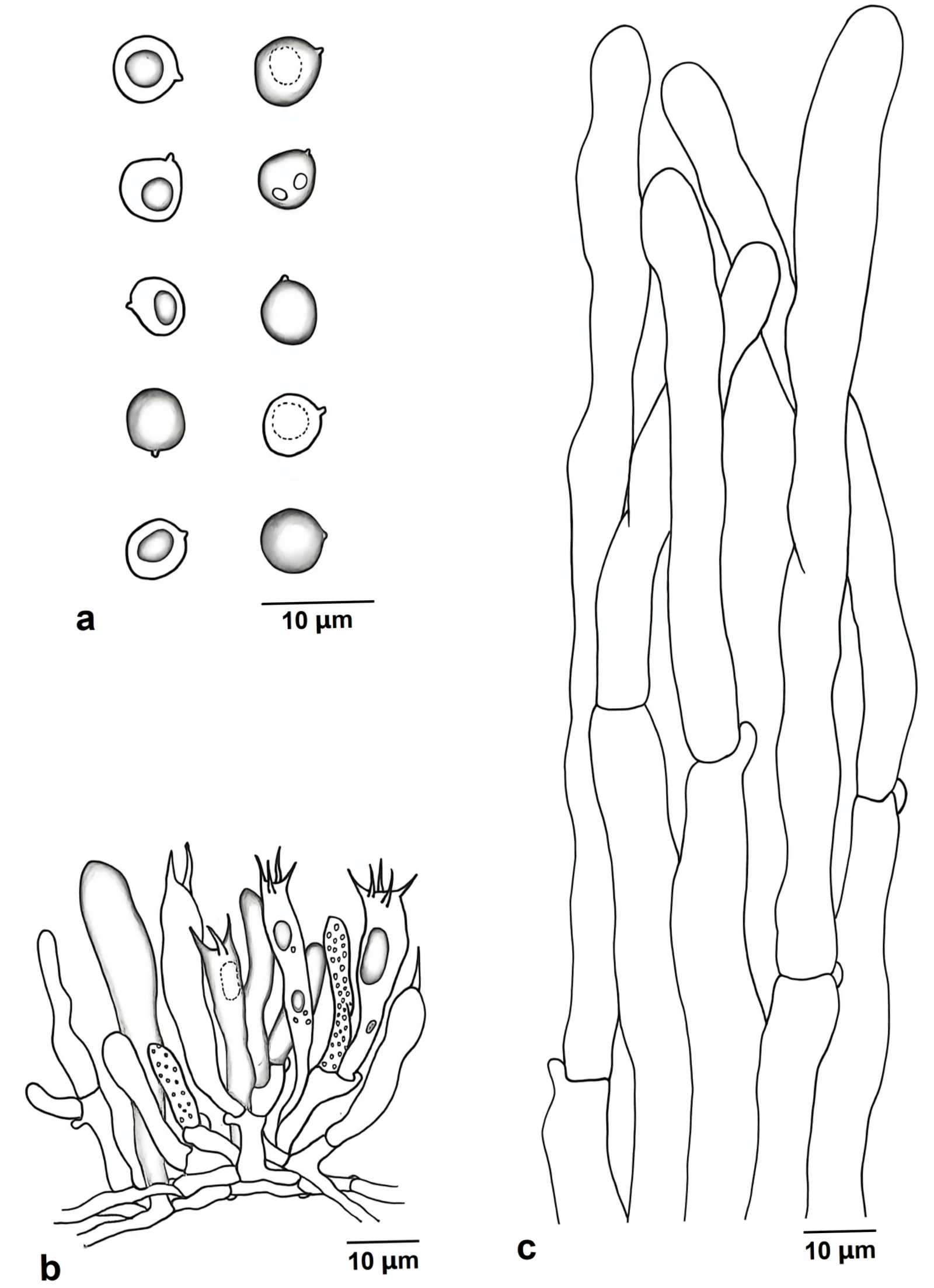
Microscopic structures of *Hydnum cavum* (holotype Yuan 19294). (**a**) Basidiospores; (**b**) Hymenium and subhymenium; (**c**) Pileipellis. Scale bar = 10 μm.

***Hydnum cavum*** T. Cao & H.S. Yuan, sp. nov. Figure 2a, Figure 3.

Mycobank: MB 864727

Etymology: *Cavum* (Lat.), refers to the hollow stipes.

Typus: China, Guangxi Province, Guilin City, on soil in *Pinus* sp. forest, 3 April 2024, H.S. Yuan, holotype, Yuan 19294 (IFP 020259).

Diagnosis: This species is characterized by its small basidiocarps, hollow stipes, small basidiospores (5.4–6.6 × 5.0–5.7 μm), relatively large basidia (30–59.4 × 6.5–15 μm) and long sterigmata up to 7 μm.

Description: Basidiocarps solitary, fleshy and leathery when fresh, becoming brittle and light in weight upon drying. Pilei 10–30 mm wide, round, convex to plano-convex, shallowly depressed in the center. Pileal surface dry, glabrous, smooth, azonate or subzonate, yellowish white or pale yellow (4A2–4A3) at center and whitish towards margin when moist. Pileal margin slightly incurved, covered with white tomentum when young. Pileal context 0.5–2 mm thick, white to yellowish white (4A1–4A2). Hymenophore hydnoid, somewhat decurrent; spines crowded, evenly distributed, fibrous, subulate, acute, straight to somewhat flexuous, solitary, 0.5–2 mm long, shortest near the pileus margin, 3–5 per mm2, brittle when dry, yellowish white (4A2) when fresh, orange to golden yellow (5A7–5B7) when dry. Stipes central, 10–30 mm long, 3–10 mm wide, subcylindrical, somewhat curved, hollow; surface glabrous, concolorous with the spine surface; stipe base enlarged. Odour mild and fruity.

Basidiospores globose, subglobose to broadly ellipsoid, (5.2–)5.4–6.6(–6.8) × (4.7–)5.0–5.7(–6.0) μm, Lm = 5.90 μm, Wm = 5.40 μm, Q = 1.01–1.22 (n = 60/2), avg.Q = 1.09, smooth, thin-walled, IKI–, hyaline, some with granular contents; hilar appendix 0.5–1 μm long. Basidia subcylindric or subclavate, 30–59.4 × 6.5–15 μm, sometimes with large guttules or finely granulose contents; sterigmata 1–5, up to 7 μm long, 1–3 μm wide at base, somewhat curving. Basidioles numerous, subcylindrical or subclavate, smaller than basidia, 12–43.5 × 3–8.5 μm. Cystidia absent. Subhymenium trama filamentous, hyphae 2–3.5 μm wide, thin-walled, olive yellow in KOH. Hyphae of spines 1.5–4 μm, thin-walled, apex cylindrical. Pileipellis composed of cylindrical hyphae, thin-walled, densely interwoven to subparallel, rarely branched; terminal elements rounded at apex, cells 46.5–131 × 7.5–20 μm. Stipitipellis composed of subcylindrical hyphae, thin-walled, interwoven, 9.5–14 μm wide, terminal elements rounded at apex. Clamp connections present.

Additional specimens examined: China, Guangxi Province, Guilin City, on soil in *Pinus* sp. forest, 3 April 2024, H.S. Yuan, paratype, Yuan 19295 (IFP 020260).

Notes: The two samples of *Hydnum cavum* cluster with *H. sp.15* [13]. The specimens are all collected from China (*H. cavum* from Guangxi and *H. sp.15* from Yunnan Province). The similarity of ITS sequences between Yuan 19294 (holotype of H. cavum, 635 bp) and HKAS55325 (553 bp) is 99.64%. The four samples (Yuan 19294, Yuan 19295; HKAS55325, HKAS92336) form a separate lineage with full support (100% ML/1.00 BPP) and we describe them as a new taxon.

**Figure 4:**
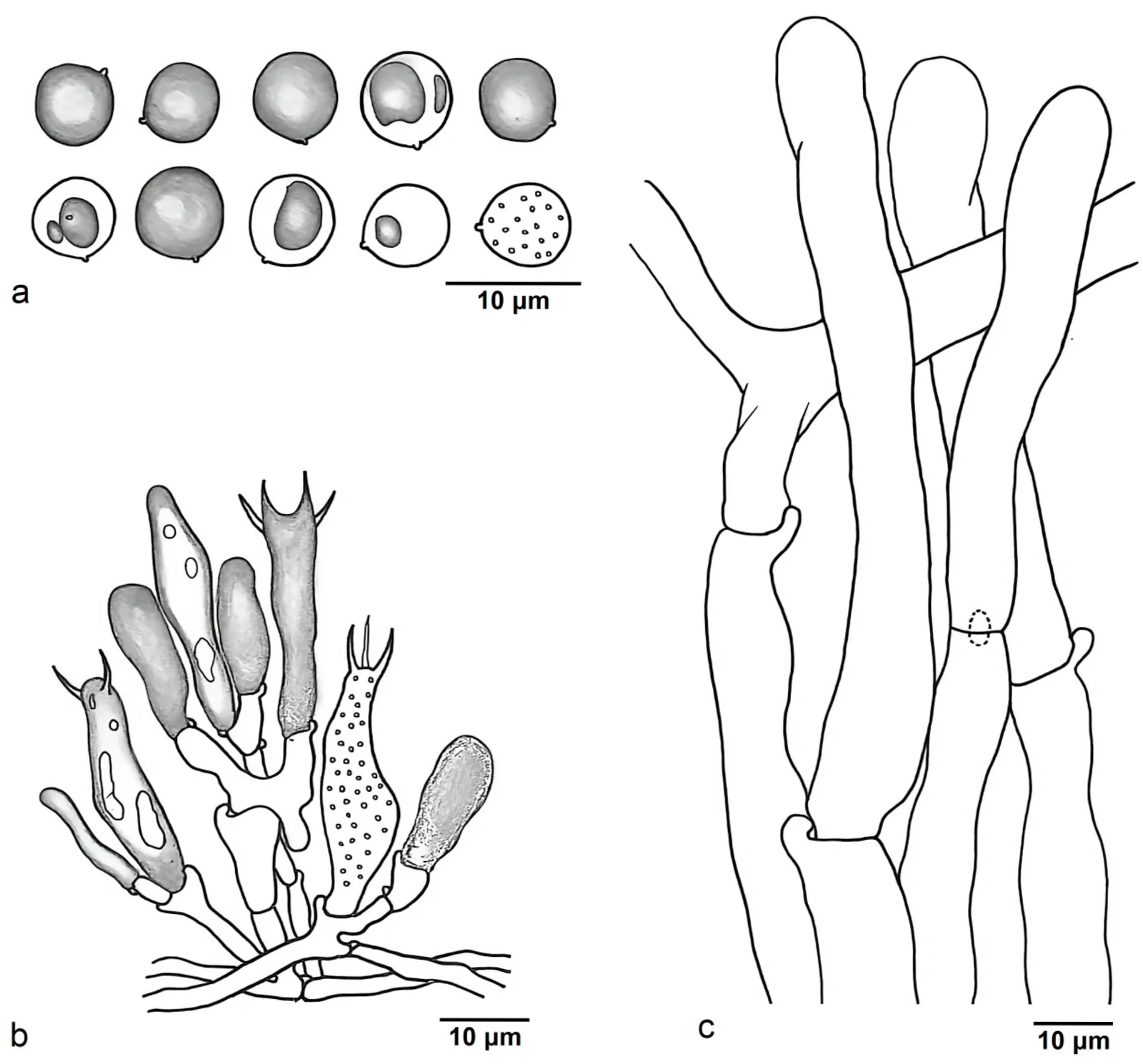
Microscopic structures of *Hydnum dazhouense* (holotype Yuan 19213). (**a**) Basidiospores; (**b**) Hymenium and subhymenium; (**c**) Pileipellis. Scale bar = 10 μm.

***Hydnum dazhouense*** T. Cao & H.S.Yuan, sp. nov. Figure 2b, Figure 4.

MycoBank: MB 864728

Etymology: *Dazhouense* (Lat.), named after the collection site of the type specimen, Dazhou City.

Typus: China, Sichuan Province, Dazhou City, Xuanhan County, on soil in mixed forest, 15 October 2023, H.S. Yuan, holotype, Yuan 19213 (IFP 020257).

Diagnosis: This species is characterized by its solitary to concrescent basidiocarps, irregularly round to flabelliform pilei, pale yellow to orange-white pileal surface, central or eccentric stipes, non-decurrent hymenophore, 2–4 sterigmata and globose, subglobose to broadly ellipsoid basidiospores.

Description: Basidiocarps solitary to concrescent, sometimes multipileate, fleshy and leathery when fresh, becoming hard and light in weight upon drying. Pilei 20–30 mm wide, irregularly round or flabelliform, depressed in the center. Pileal surface dry, glabrous, smooth, pale yellow to orange-white (4A3–5A2) when dry. Pileal margin thin, entire to incised, straight or slightly decurved, concolorous with the pileal surface. Pileal context 1–2 mm thick, yellowish white to pale yellowish white (1A2–2A2). Hymenophore hydnoid, adnate, non-decurrent, surface orange-white (4A3) upon drying; spines fibrous, subulate, acute, straight to somewhat flexuous, solitary, 1–3 mm long, 2–3 per mm^2^, brittle when dry. Stipes central or eccentric, 10–25 mm long, 3–10 mm wide, subcylindrical, solid; surface glabrous, concolorous with the spine surface, staining brownish orange when handled; stipe base slightly enlarged. Odour mild and fruity.

Basidiospores globose, subglobose to broadly ellipsoid, (5.0–)6.0–7.9(–8.8) × (5.2–)5.9–7.6(–7.9) μm, Lm = 7.34 μm, Wm = 6.77 μm, Q = 1.01–1.25 (n = 60/2), avg.Q = 1.08, smooth, thin-walled, IKI–, hyaline, some with granular contents; hilar appendix up to 1 μm long. Basidia subcylindric, subclavate to clavate, 31–46.5 × 5.5–11 μm, sometimes with finely granulose contents; sterigmata 2–4, up to 5 μm long, 1–3 μm wide at base, somewhat curving. Basidioles numerous, subcylindrical or subclavate, smaller than basidia, 14.5–45 × 3–10.5 μm. Cystidia absent. Subhymenium trama filamentous, hyphae 1.5–5 μm wide, thin-walled, olive yellow in KOH. Hyphae of spines 2–6.5 μm, thin-walled, apex cylindrical. Pileipellis composed of cylindrical hyphae, thin-walled, densely interwoven to subparallel, often branched; terminal elements rounded at apex, cells 75–128 × 8–21.5 μm. Stipitipellis composed of subcylindrical hyphae, thin-walled, interwoven, 8–14.5 μm wide, terminal elements rounded at apex. Clamp connections present.

Additional specimens examined: China, Sichuan Province, Dazhou City, Xuanhan County, on soil in mixed forest, 15 October 2023, H.S. Yuan, paratype, Yuan 19214 (IFP 020258).

Notes: *Hydnum dazhouense* is closely related to *H. tangerinum* in the tree. Meanwhile, the samples of *H*. *dazhouense* and *H. tangerinum* are all collected from subtropical forest in China. In terms of morphology characteristics, the new species resembles the latter one in having solitary to concrescent basidiocarps, irregularly round to flabelliform pilei, orange-white tint pileal surface, central or eccentric stipes and non-decurrent hymenophore, but differs by having smaller basidiocarps (pilei up to 30 mm vs. 50 mm wide; stipes up to 25 mm vs. 60 mm long), shorter spines (1–3 mm vs. 2–6 mm long), subglobose basidiospores (avg.Q = 1.08 vs. 1.31), 2–4 sterigmata, broader hyphae of spines (2–6.5 μm vs. 2–3.5 μm), shorter terminal elements cells of pileipellis and thin-walled stipitipellis [9].

**Figure 5:**
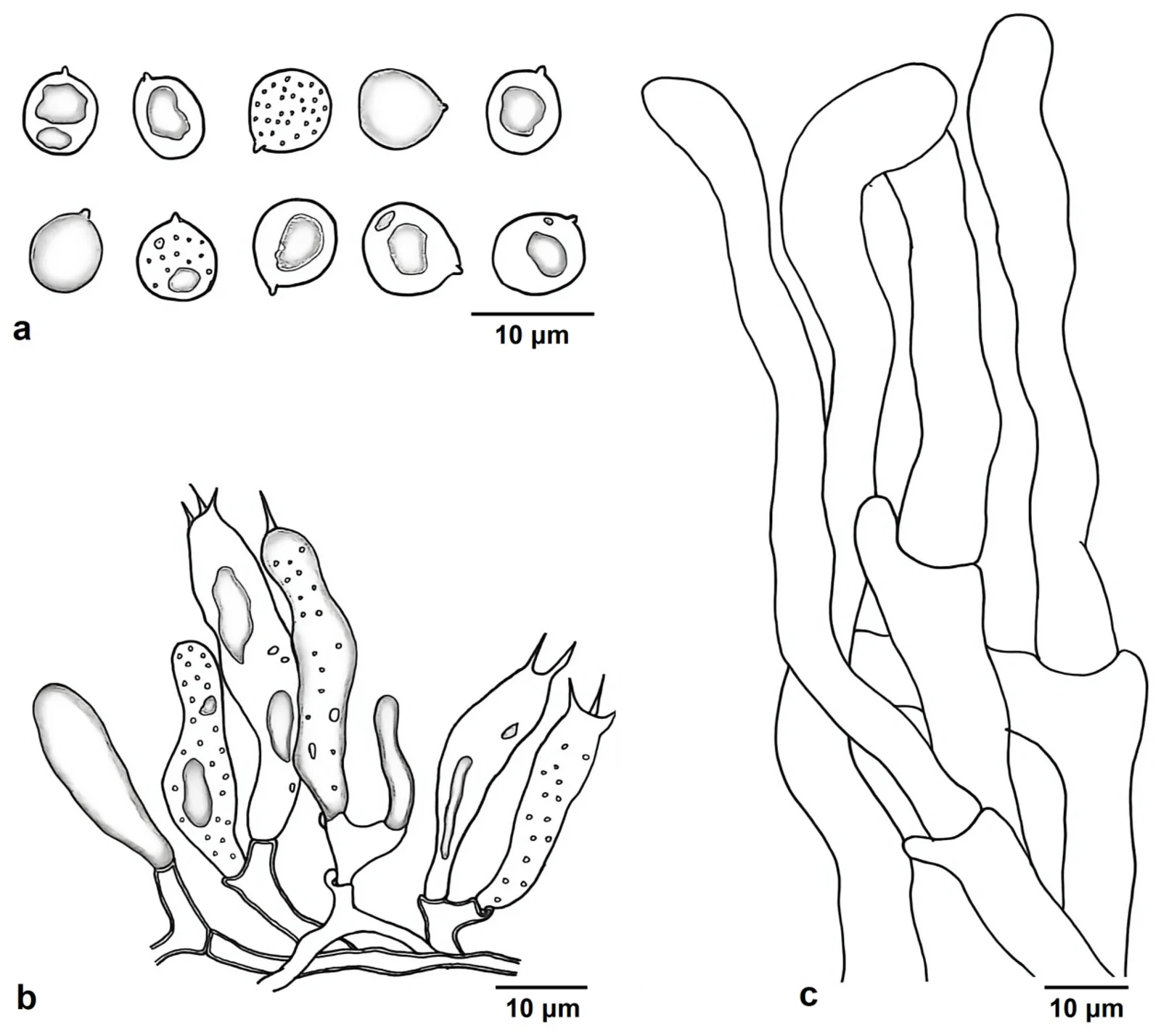
Microscopic structures of *Hydnum albopallidum* (CFSZ 12790). (**a**) Basidiospores; (**b**) Hymenium and subhymenium; (**c**) Pileipellis. Scale bar = 10 μm.

***Hydnum albopallidum*** R. Sugaw. & N. Endo. Figure 2c, Figure 5.

Notes: *Hydnum albopallidum* is originally described from Japan and firstly recorded in China in our study. Morphologically, it is characterized by small to medium basidiocarps, whitish pileal surface, subdecurrent spines, subglobose to broadly ellipsoid basidiospores (Qm = 1.14–1.19), 2–4 sterigmata and thin-walled hyphae in hymenium and rhizomorphs [14]. The micromorphological features observed in this study are generally similar to those in the original description. Nevertheless, upon examination of the sterigmata, it was observed that single sterigma occurs in this species (Figure 5). It is somewhat at variance with the original description. Phylogenetically, the Chinese specimen is well nested within the branch of *H. albopallidum* with a strong support (99% ML/1.00 BPP). The specimens from both countries are all located in the temperate zone, but their hosts are slightly different (in angiosperm and *Pinus* sp. mixed forest for CFSZ12790 vs. *Abies* sp., *Betula* sp., *Tsuga* sp. and *Alnus* sp. for previous studies).

Specimens examined: China, Inner Mongolia, Chifeng City, Kalaqin Banner, Wangyezhen Daxigou, on soil in angiosperm and *Pinus* sp. mixed forest, 14 July 2017, T.Z. Liu, CFSZ12790 (IFP 020263).

**Figure 6:**
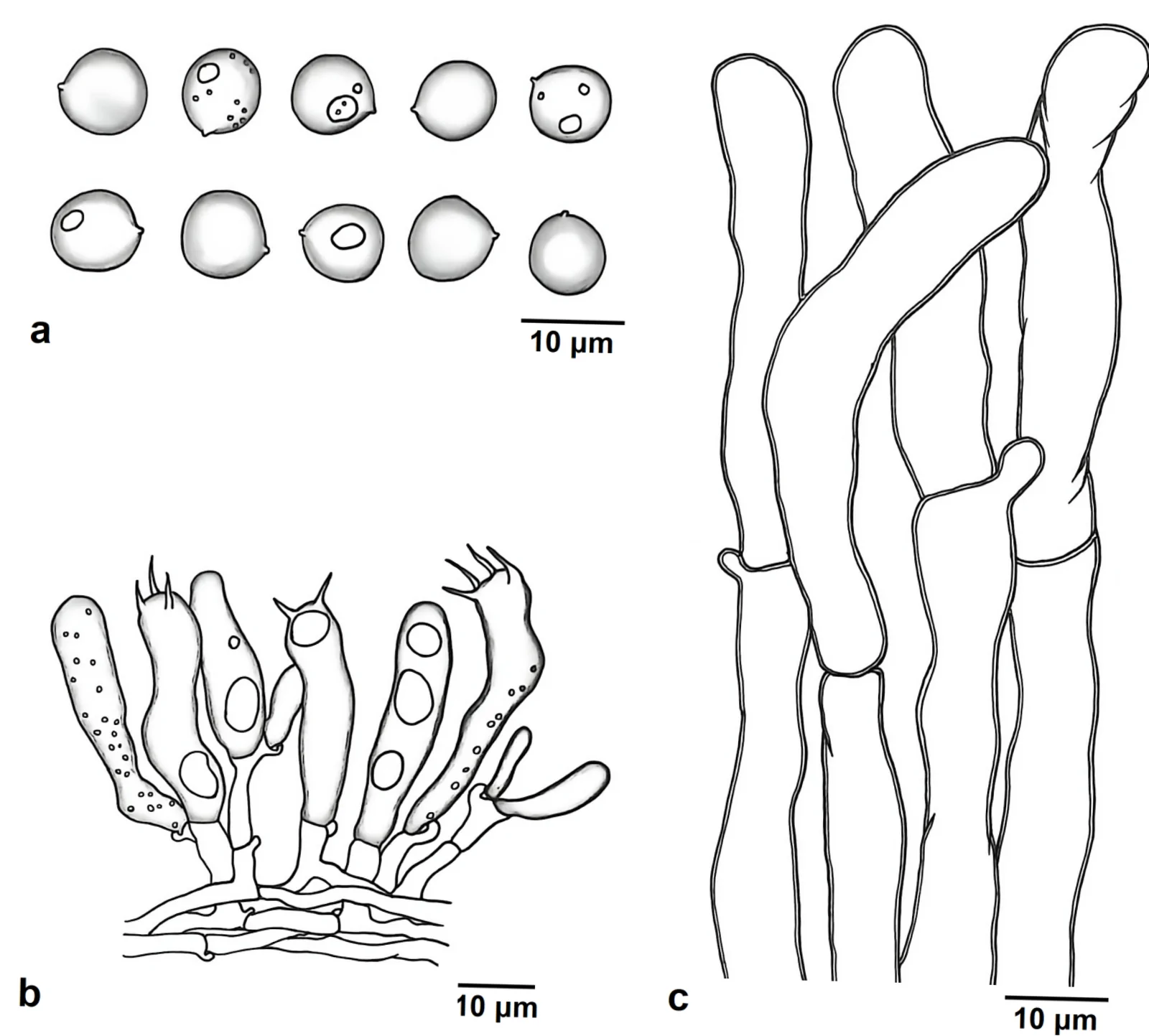
Microscopic structures of *Hydnum repando-orientale* (Yuan 20830). (**a**) Basidiospores; (**b**) Hymenium and subhymenium; (**c**) Pileipellis. Scale bar = 10 μm.

***Hydnum repando-orientale*** Liimat. & Niskanen. Figure 2d, Figure 6.

Notes: Phylogenetic and morphological analyses based on ITS, nLSU and *tef*-*1α* sequences confirmed the new record. To date, this species has only been collected in two East Asian countries, China and Japan. Phylogenetically, the specimens of *H. repando-orientale* from China, Yunan and Gansu Province, form a strong support (100% ML/1.00 BPP) branch with the holotype. Meanwhile, the branch are closely related to *H. boreorepandum* Niskanen, Liimat. & Niemelä, *H. neorepandum* Niskanen & Liimat., *H. repandum* L., *H. sinorepandum* Ming Zhang & C.Q. Wang, *H. sphaericum* Ting Cao & H.S. Yuan, *H. vagabundum* Swenie, Ovrebo & Matheny and *H. washingtonianum* Ellis & Everh. which are all nested in the Sect. *Hydnum* and belong to Subgen. *Hydnum*. This species was originally described as *H. repandum* var. *album* (Quél.) Rea from Japan by Yanaga et al. [12]. In 2018, Niskanen et al. [1] revised it to *H. repando-orientale*. Morphologically, *H. repando-orientale* characterized by its relatively large basidiocarps, yellowish white or greyish yellow pileal surface, decurrent hymenophore, orange-white and sometimes bifurcated spines, 2–4 sterigmata and large basidiospores (7.2–9.1 × 6.4–8.3 μm). No major difference was found between Chinese and Japanese specimens. Given the original description provided for the images of basidiocarps and baisidiospores, we have supplemented detailed line drawings of the microscopic characters for this species.

Specimens examined: China, Yunnan Province, Shangri-La City, Xiaozhongdian Town, Pinghushan Mt., on soil in *Quercus* sp. and *Pinus* sp. mixed forest, 4 September 2024, H.S. Yuan, Yuan 20830 (IFP 020261); Gansu Province, Tianshui City, on soil in mixed forest, 3 September 2024, H.S.Yuan, Yuan 20662 (IFP 020262).

Key to Species of *Hydnum* in China
1. Basidiomata whitsh or pale yellow
21. Basidiomata yellow to orange
162. Pileus white
32. Pileus pale yellow
73. Pileus ≤ 30 mm wide
*H. flavidocanum*3. Pileus ≥ 30 mm wide
44. Habitat in broad-leaved and mixed forests
*H. cremeoalbum*4. Habitat in broad-leaved forests
55. Pileus ≤ 45 mm wide
65. Pileus up to 57 mm wide
*H. treui*6. Basidiospores ≤ 4 μm wide on average
*H. minus*6. Basidiospores > 4 μm wide on average
*H. orientalbidum*7. Pileus up to 60 mm wide
87. Pileus ≤ 60 mm wide
108. Habitat in broad-leaved forests
98. Habitat in mixed forests
*H. repando-orientale*9. Spines yellowish white
*H. bifurcatum*9. Spines pale orange
*H. roseoalbum*10. Habitat in broad-leaved forests
1110. Habitat in mixed or *Pinus* sp. forests
1211. Spines > 6 mm long
*H. albomagnum*11. Spines < 6 mm long
*H. brevispinum*12. Baisidiospores ≤ 5 μm wide on average
1312. Baisidiospores ≥ 5 μm wide on average
1413. Habitat in mixed forests
*H. robustum*13. Habitat in Pinus sp. forests
*H.pinicola*14. Basidiocarps ≤ 30 mm on wide
*H. cavum*14. Basidiocarps > 30 mm on wide
*H. albopallidum*15. Basidiomata yellowish white
1615. Basidiomata orange
2216. Basidiospores < 6 μm long on average
*H. microcarpum*16. Basidiospores > 6 μm long on average
1717. Basidiospores < 7.5 μm long on average
1817. Basidiospores > 7.5 μm long on average
2118. Pileus > 40 mm wide
*H. fulvostriatum*18. Pileus < 40 mm wide
1919. Spines > 3 mm long
*H. luteoalbum*19. Spines ≤ 3 mm long
2020. Sterigmata 2–6
*H. tenuistipitum*20. Sterigmata 2–4
*H. albotomentosum*21. Habitat in *Q. variabilis* forests
*H. crassipedum*21. Habitat in *Q. serrata* forests
*H. albomarginatum*22. Basidiospores < 8 μm long on average
2322. Basidiospores > 8 μm long on average
2723. Pileus ≥ 60 mm wide
2423. Pileus < 60 mm wide
2524. Habitat in coniferous forests
*H. jussii*24. Habitat in forest mixed by *Bamboo*, *Quercus* and *Pinus*
*H. flosculoides*25. Pileus brownish orange to light brown
*H. albodentum*25. Pileus pale yellow
2626. Pileipellis hyphae thin-walled
*H. dazhouense*26. Pileipellis hyphae slightly thick-walled
*H. erectum*27. Basidiospores 8–9 μm long on average
2827. Basidiospores > 9 μm long on average
3528. Spines white
2928 Spines orange-white or cream-yellow
3029. Spines ≤ 3 mm long
*H. sphaericum*29. Spines ≥ 3 mm long
*H. sinorepandum*30. Spines cream-yellow
*H. cremeum*30. Spines orange-white to brownish orange
3131. Basidiospores 8.1–8.5 μm long on average
3231. Basidiospores > 8.5 μm long on average
3432. Spines < 2 mm long
*H. vesterholtii*32. Spines > 2 mm long
3333. Habitat in angiosperm forests
*H. tangerinum*33. Habitat in *Q. serrata* forest
*H. berkeleyanum*34. Basidiospores Q < 1.1
*H. ventricosum*34. Basidiospores Q > 1.1
*H. pallidomarginatum*35. Pileus < 50 mm wide
3635. Pileus > 50 mm wide
*H. roseotangerinum*36. Spines orange-white to pale orange
3736. Spines light-yellow to pale orange
3937. Pileus ≥ 30 mm wide
*H. flabellatum*37. Pileus ≤ 30 mm wide
3838. Basidiospores > 9.5 μm long on average
*H. longibasidium*38. Basidiospores < 9.5 μm long on average
*H. longipes*39. Basidiospores Q > 1.3
*H. pallidocroceum*39. Basidiospores Q < 1.3
4040. Pileus ≤ 40 mm wide
*H. melitosarx*40. Pileus > 40 mm wide
*H. flavoquamosum*

## 4. Discussion

This study documents two new *Hydnum* species together with two new record species from China. Molecular and morphological evidence clearly distinguishes the two new species, namely *H. cavum* and *H. dazhouense*, from other known species. Two new record species form a high-supported branch with *H. albopallidum* and *H. repando-orientale* respectively and shared highly similar morphological characteristics with the type specimen.

In recent years, new species of *Hydnum* have been continuously discovered. In just the two-year period of 2025 and 2026, a total of 13 new species have been described worldwide, among which nine species are from China. Among them, *H. albomarginatum* Y.L. Tuo, B. Zhang & Y. Li, *H. crassipedum* Y.L. Tuo, B. Zhang & Y. Li, *H. fulvostriatum* Y.L. Tuo, B. Zhang & Y. Li and *H. longipes* Ming Zhang & C.Q. Wang are all nested in subg. *Rufescentia* and closely related to *H. dazhouense*. However, *H. dazhouense* differs from *H. albomarginatum* and *H. crassipedum* in having a smaller pileus (20–30 vs. 23–66 vs. 24–56 mm wide), and smaller (7.34 × 6.77 vs. 8.07 × 7.92 vs. 8.0 × 6.99 µm) and slender (Q = 1.01–1.25 vs. 1.00–1.09 vs. 1.11–1.17) basidiospores [16]. *H. fulvostriatum* can be distinguished from *H. dazhouense* by the larger pileus (44–65 mm wide) and boarder basidiospores (Q = 1.00–1.08) [27], and *H. longipes* differs from the new species by having the orange-to-reddish orange pileus, longer stipe as well as larger basidiospores (9.37 × 8.71 µm) [15].

In the phylogenetic tree, eight clades with moderate to strong support were obtained. Several subgenera of *Hydnum*, namely *Alba*, *Pallida*, *Hydnum*, *Rufescentes*, *Alba s.l*., *Brevispina* as well as the *H. orientalbidum* R. Sugaw. & N. Endoclade clade are phylogenetically consistent with the results of previous studies. The new species *H. cavum* and *H. sp.15* constitute a new branch which is clearly distinct from any other subgenera. *H. cavum* is characterized by its yellowish white or pale yellow pilei, small basidiocarps, hollow stipes, small basidiospores (5.4–6.6 × 5.0–5.7), relatively large basidia (30–59.4 × 6.5–15 μm) and long sterigmata up to 7 μm. The species in subg. *Alba* resembles *H. cavum* in having small basidiospores (on av. <7 × 7 μm) and whitish pileus, but they can be differentiated from the new species by having solid stipes [1,14,17]. The species in subg. *Pallida* is closely related to *H. cavum* by having small basidiocarps but differs from the latter by having slender basidiospores (Qm usually >1.25 vs. 1.01–1.22). Subg. *Hydnum* can be distinctly differentiated from *H. cavum* in having medium to large (pileus (30–)40–110 mm diam) and larger basidiospores (7–9.2 × 5.5–8 μm), while Subg. *Rufescentia* can be identified by its pale ochraceous brown to orange ochraceous pileus [1]. Several species in subg. *Brevispina,* including *H. alboaurantiacum* Swenie & Matheny, *H. alboluteum* R. Sugaw. & N. Endo, *H. brevispinum* Ting Cao & H.S. Yuan, *H. microcarpum* Ming Zhang and *H. tenuistipitum* Ting Cao & H.S. Yuan, all have solid stipes which can be clearly distinguished from the hollow stipes of *H. cavum* [4,9,14,15]; stipes of *H. minus* Yanaga & N. Maek are solid when young and hollow in age, but it differs from *H. cavum* by having small (4.72 × 3.68 µm) and slender basidiospores (Qm = 1.29) [12,25]. However, more researchable species are still needed to determine the stable morphological synapomorphies of specimens in order to support the branch as a new subgenus.

*Hydnum albopallidum* was originally described in Japan [14]. In this study, the Chinese specimen CFSZ12790 and the type TUMH 63997, as well as TUMH 63998, grouped together in the same branch which received a strong support (99% ML/1.00 BPP). Feng et al. [13] discovered two specimens, HKAS92341 and HKAS92343 in Shaanxi Province of China. These two specimens were placed in the *Vesterholtii* clade and were named *H. vesterholtii* Olariaga, Grebenc, Salcedo & M.P. Martín (RE2). Baroni et al. [42] included the ITS fragment of HKAS92341 in the ML phylogenetic tree. It was indeed grouped together with the type specimen TUMH63997, but the support rate was only 70 in ML. The similarity between CFSZ12790 and HKAS92341 as well as HKAS92343 is 96.99% and 97.30%, respectively. Therefore, whether HKAS92341 and HKAS92343 are *H. albopallidum* or cryptic species within this species complex still requires further verification by combining morphological characteristics and more molecular information.

*Hydnum repando-orientale* specimen Yuan 20662 and Yuan 20830, together with the type specimen TUMH 60745 and TUMH 60743, form a branch with a full support (100% ML/1.00 BPP). The morphological characteristics are not much different from the original description. However, there are differences between our specimens and the type in terms of habitat: *H. repando-orientale* distributed in the temperate and cold temperate regions of China (Yuan 20662 were collected from Tianshui, Gansu Province, and Yuan 20830 from Xiaozhongdian, Shangri-La of Yunnan Province), while TUMH 60745 and TUMH 60743 were both located in temperate regions of Japan. Furthermore, *H. repando-orientale* is currently documented exclusively in coniferous and broad-leaved mixed forests in China, whereas its distribution in Japan is broad-leaved forests [1]. The “low altitude and broad-leaved tree” preference of *H. repando-orientale* may represent more primitive ecological niche of this species. The differences in habitat between specimens of China and Japan actually represent an ecological differentiation that occurred after an ancestral species spread to China and adapted to different altitudes and hosts. It is likely an ongoing ecological speciation event.

Recent comprehensive phylogenetic and biogeographic analyses have increasingly clarified the global distribution patterns of *Hydnum*. The *Hydnum* flora of China is of particular interest because it comprises both endemic lineages (such as the new species *H. cavum* and *H. dazhouense* described herein) and more broadly distributed East Asian species (such as the newly recorded *H. albopallidum* and *H. repando-orientale*), reflecting complex interactions among speciation, dispersal, and allopatric speciation. This study enriches the biodiversity of the genus *Hydnum* in China and contributes to a broader understanding of the biogeographic history of this ecologically important genus of ectomycorrhizal fungi.

## Figures and Tables

**Table 1:** Specimens and sequences used in this study.

Species	GenBank No.			Specimen/Culture Voucher	Country
	ITS	nLSU	*tef*-*1α*		
*H. albertense*	KX388664	-	-	H T. Niskanen 11-354(HT)	Canada
*H. albomagnum*	DQ218305	AY700199	DQ234568	AFTOL-ID 471	USA
*H. albomagnum*	MH379943	-	-	RAS231(epi)	USA
*H. albomarginatum*	PV329855	PV356813	-	FJAU66574	China
*H. albomarginatum*	PV329856	PV356814	-	FJAU66575	China
*H. albopallidum*	NR_176696	LC717904	LC622442	TUMH 63997(T)	Japan
*H. albopallidum*	LC621808	-	LC622443	TUMH 63998	Japan
***H. albopallidum***	**PZ734842**	**PZ734853**	**PZ798148**	**CFSZ12790**	**China (This study)**
*H. albotomentosum*	PV329851	-	-	FJAU66568 (T)	China
*H. albotomentosum*	PV329852	-	-	FJAU66569	China
*H. atlanticum*	OQ235218	-	-	AJ1597	Canada
*H. atlanticum*	OQ235214	-	-	AJ1598	Canada
*H. berkeleyanum*	NR158533	NG070500	-	CAL 1656(T)	India
*H. berkeleyanum*	MW980552	MW979538	-	Wei 10375	China
*H. bifurcatum*	PV329845	-	PP357252	FJAU66562	China
*H. bifurcatum*	PV329846	-	PP357253	FJAU66563	China
*H. boreorepandum*	KX388658	-	-	H T. Niemela 1679	Finland
*H. boreorepandum*	KX388657	-	-	H 6003711(T)	Finland
*H. brevispinum*	MW980578	MW979559	-	Wei 10214(T)	China
*H. brevispinum*	MW980579	MW979560	-	Wei 10258	China
*H. canadense*	KX388681	-	-	H T. Niskanen 09-006(T)	Canada
***H. cavum***	**PZ734843**	**PZ734854**	**PZ798149**	**Yuan 19294(T)**	**China (This study)**
***H. cavum***	**PZ734844**	**PZ734855**	**PZ798150**	**Yuan 19295**	**China (This study)**
*H. cremeoalbum*	AB906674	-	-	TUMH 40462	Japan
*H. cremeoalbum*	AB906678	-	-	TUMH 60740(HT)	Japan
*H. cuspidatum*	MH379944	-	-	RAS 246(T)	USA
*H. cuspidatum*	MH379936	-	-	RAS 205	USA
***H. dazhouense***	**PZ734838**	**PZ734849**	**-**	**Yuan 19213(T)**	**China (This study)**
***H. dazhouense***	**PZ734839**	**PZ734850**	**-**	**Yuan 19214**	**China (This study)**
*H. ellipsosporum*	AY817138	-	-	Os5579(T)	Germany
*H. ellipsosporum*	KX388671	-	-	HTN 12036	Finland
*H. ferruginescens*	MH379905	-	-	MH16005(T)	USA
*H. ferruginescens*	MH379942	-	-	RAS229	USA
*H. flabellatum*	MW980575	MW979556	-	Yuan 14708 (T)	China
*H. flosculoides*	PQ505047	PQ505039	-	Cui 22857	China
*H. fulvostriatum*	PV329849	PV356807	-	FJAU66566	China
*H. fulvostriatum*	PV329850	PV356808	-	FJAU66567	China
*H. jussii*	KX388665	-	-	H 6003709(T)	Finland
*H. jussii*	MW980553	MW979539	MW999436	Yuan 14008	China
*H. jussii*	MW980554	MW979540	MW999437	Yuan 14009	China
*H. luteoalbum*	PV329847	-	-	FJAU66564 (T)	China
*H. luteoalbum*	PV329848	-	-	FJAU66565	China
*H. magnorufescens*	KU612549	KU612669	-	voucher 161209	Slovenia
*H. magnorufescens*	KC293545	-	-	TO HG2818(T)	Italy
*H. melitosarx*	KX388683	-	-	H 7043937(T)	USA
*H. melitosarx*	OR947117	OR947136	OR944614	GDGM84518	China
*H. melitosarx*	OR947125	OR947142	OR944619	GDGM81873	China
*H. melleopallidum*	FJ845406	-	-	SMI356(T)	Canada
*H. microcarpum*	NR_198673	OR947134	OR944612	GDGM87902	China
*H. microcarpum*	PQ505069	PQ505033	PX148769	Cui 23219	China
***H. microcarpum***	**PZ734845**	**PZ734856**	**-**	**Yuan 17652**	**China (This study)**
***H. microcarpum***	**PZ734846**	**PZ734857**	**-**	**Yuan 17653**	**China (This study)**
*H. minospororufescens*	NR176699	-	LC622467	TUMH 64041(T)	Japan
*H. minospororufescens*	LC621835	-	LC622466	TUMH 64039	Japan
*H. mulsicolor*	AJ547885	-	-	LJU GIS 1336(T)	Slovenia
*H. mulsicolor*	JX093560	-	-	REB341	USA
*H. neorepandum*	KX388659	-	-	H T. Niskanen 10-095(T)	Canada
*H. neorepandum*	KX388660	-	-	H T. Niskanen 10-086	Canada
*H. olympicum*	KX388661	-	-	09-134(T)	USA
*H. olympicum*	MT955159	-	-	SAT-10-208-05	USA
*H. oregonense*	KF879509	-	-	HVM61	USA
*H. oregonense*	AJ534972	-	-	PNW-MS g2010502h1-09 (T)	USA
*H. orientalbidum*	PV329857	PV356809	-	FJAU66570	China
*H. orientalbidum*	PV329858	PV356810	-	FJAU66571	China
*H. ovoideisporum*	KU612536	-	-	voucher 71106	Slovenia
*H. ovoideisporum*	NR119818	-	-	BIO Fungi 12683 (T)	Spain
*H. pallidocroceum*	MW980568	MW979554	MW999449	Yuan 14023	China
*H. pallidocroceum*	MW980569	MW979555	MW999450	Yuan 14017	China
*H. pallidomarginatum*	MW980566	MW979552	MW999447	Yuan 13928a (T)	China
*H. pallidomarginatum*	MW980567	MW979553	MW999448	Yuan 13940a	China
*H. pinicola*	OR211383	OR211401	OR220059	SFC20180928-18	Korea
*H. quebecense*	KX388662	-	-	HTN 10064 (T)	Canada
*H. quebecense*	MH379881	-	-	CN9	USA
*H. repandum*	NR164553	-	-	H6003710(T)	Finland
*H. repando-orientale*	AB906683	-	-	TUMH60745(T)	Japan
*H. repando-orientale*	AB906684	-	-	TUMH:60743	Japan
***H. repando-orientale***	**PZ734840**	**PZ734851**	**PZ798146**	**Yuan 20830**	**China (This study)**
***H. repando-orientale***	**PZ734841**	**PZ734852**	**-**	**Yuan 20662**	**China (This study)**
*H. robustum*	PQ505050	PQ505042	-	Cui 22893 (T)	Finland
*H. robustum*	PQ505048	PQ505040	-	Cui 22901	Finland
*H. rufescens*	KX388688	-	-	H 6003708(epi)	Finland
*H. rufescens*	KX388656	-	-	HTN7839	Estonia
*H. sinorepandum*	NR_198675	OR947139	OR944616	GDGM 82445(T)	China
*H. sinorepandum*	OR947120	OR947138	OR944615	GDGM82416	China
***H. sinorepandum***	**PZ734847**	**PZ734858**	**-**	**Yuan 20299**	**China (This study)**
***H. sinorepandum***	**PZ734848**	**PZ734859**	**-**	**Yuan 20304**	**China (This study)**
*H. slovenicum*	AJ547870	-	-	LJU GIS 1338(T)	Slovenia
*H. slovenicum*	AJ547884	-	-	LJU GIS 1340	Slovenia
*H. sp.1*	KC679834	-	-	wi1A4spel	Taiwan
*H. sp.1*	KC679833	-	-	wi8T4spel	Taiwan
*H. sp.13*	KU612617	KU612673	-	HKAS57714	China
*H. sp.13*	KU612616	KU612675	-	HKAS58838	China
*H. sp.15*	KU612613	-	-	HKAS55325	China
*H. sp.15*	KU612614	-	-	HKAS92336	China
*Hy. sphaericum*	MW980563	MW979549	MW999444	Wei 10243 (T)	China
*Hy. sphaericum*	MW980564	MW979550	MW999445	Wei 10300	China
*Hy. sphaericum*	MW980565	MW979551	MW999446	Wei 10262	China
*H. subberkeleyanum*	LC621879	-	-	TNS:F-19323	Japan
*H. subberkeleyanum*	LC621880	-	LC622505	TUMH 63627	Japan
*H. subconnatum*	MH379930	-	-	RAS235(T)	USA
*H. subconnatum*	MH379916	-	-	RAS169	USA
*H. subcremeoalbum*	UDB013289	-	-	TU110688 (T)	Papua New Guinea
*H. submulsicolor*	KX388682	-	-	H T. Niskanen 10-132(T)	Canada
*H. subolympicum*	KU612599	KU612653	-	F1188765	USA
*H. subolympicum*	MH174257	-	-	DAOM744368(T)	Canada
*H. subovoideisporum*	NR158494	-	-	H 6003707(T)	Finland
*H. subrufescens*	KX388649	-	-	H T. Niskanen 10-154(T)	Canada
*H. subrufescens*	KU612535	KU612663	-	F1188749	USA
*H. subtilior*	MH379918	-	-	RAS180	USA
*H. subtilior*	NR164029	-	-	TENN073034(T)	USA
*H. tangerinum*	MW980580	MW979561	-	Wei 10245(T)	China
*H. tangerinum*	MW980581	MW979562	-	Wei 10249	China
*H. tangerinum*	MW980582	MW979563	-	Wei 10250	China
*H. tenuistipitum*	MW980576	MW979557	-	Wei 10410(T)	China
*H. tenuistipitum*	MW980577	MW979558	-	Wei 10417	China
*H. tottoriense*	LC621887	-	LC622511	TUMH64088	Japan
*H. tottoriense*	LC621888	-	LC622512	TUMH64089	Japan
*H. treui*	UDB013043	-	-	TU110403 (T)	Papua New Guinea
*H. vagabundum*	MH379909	-	-	CLO4985(T)	USA
*H. vagabundum*	MH379949	-	-	10782TJB	USA
*H. ventricosum*	MW980561	MW979547	MW999442	Yuan 14536	China
*H. ventricosum*	MW980562	MW979548	MW999443	Yuan 14601	China
*H. washingtonianum*	MF954990	-	-	UBC F-32538	Canada
*H. zongolicense*	KC152121	-	-	GO-2010-142a (T)	Mexico
*Sistotrema muscicola*	AJ606040	AJ606040	-	KHL 11721	Sweden
*S*. *muscicola*	AJ606041	-	-	taxon:154757	Sweden

Note: “-”, not applicable; Newly generated sequences in this study are in bold; type specimens are marked with “T”.

## Data Availability

The sequences from the present study were submitted to the NCBI website (https://www.ncbi.nlm.nih.gov/, accessed on 25 July 2026), and the accession numbers were listed in Table 1.
